# Supplementary material for: Purification and Glutaraldehyde Activation Study on HCl-Doped PVA–PANI Copolymers with Different Aniline Concentrations
Source: Molecules. 2018 Dec 25;24(1):63. doi: 10.3390/molecules24010063 (PMC6337460; doi:10.3390/molecules24010063)
Supplement: Supplementary file 1 [file molecules-24-00063-s001.pdf]

Supplementary material

Purification and Glutaraldehyde Activation Study on HCl-Doped PVA–PANI Copolymers with Different Aniline Concentrations

Jorge M. Guerrero [1], Amanda Carrillo [2,*], María L. Mota [2,3], Roberto C. Ambrosio [4] and Francisco S. Aguirre [1,*]

[1] Centro de Investigación en Materiales Avanzados, S.C., Alianza Norte 202, Parque de Investigación e Innovación Tecnológica, Apodaca, NL, C.P. 66600, México; jorge.guerrero@cimav.edu.mx (J.M.G); servando.aguirre@cimav.edu.mx (F.S.A)

[2] Instituto de Ingeniería y Tecnología, Universidad Autónoma de Ciudad Juárez, Av. Del Charro 610, Ciudad Juárez, CHIH, C.P. 32310, México; amanda.carrillo@uacj.mx (A.C.); mdllmotago@conacyt.mx (M.L.M.)

[3] CONACYT, Universidad Autónoma de Ciudad Juárez, Ciudad Juárez, CHIH, C.P. 32310, México

[4] Facultad de Electrónica, Benemérita Universidad Autónoma de Puebla, Puebla, C.P 72000, México; roberto.ambrosio@correo.buap.mx (R.C.A.)

* Correspondence: amanda.carrillo@uacj.mx, Tel:+52-1-656-688-4800; servando.aguirre@cimav.edu.mx; Tel.: +52-1-811-721-6676

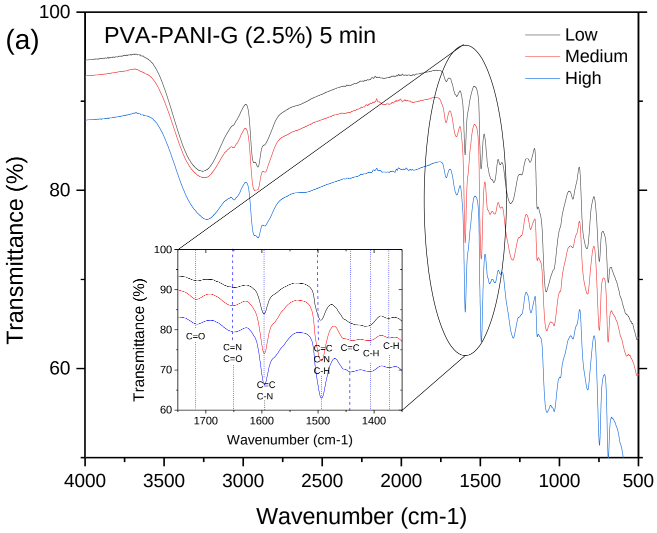

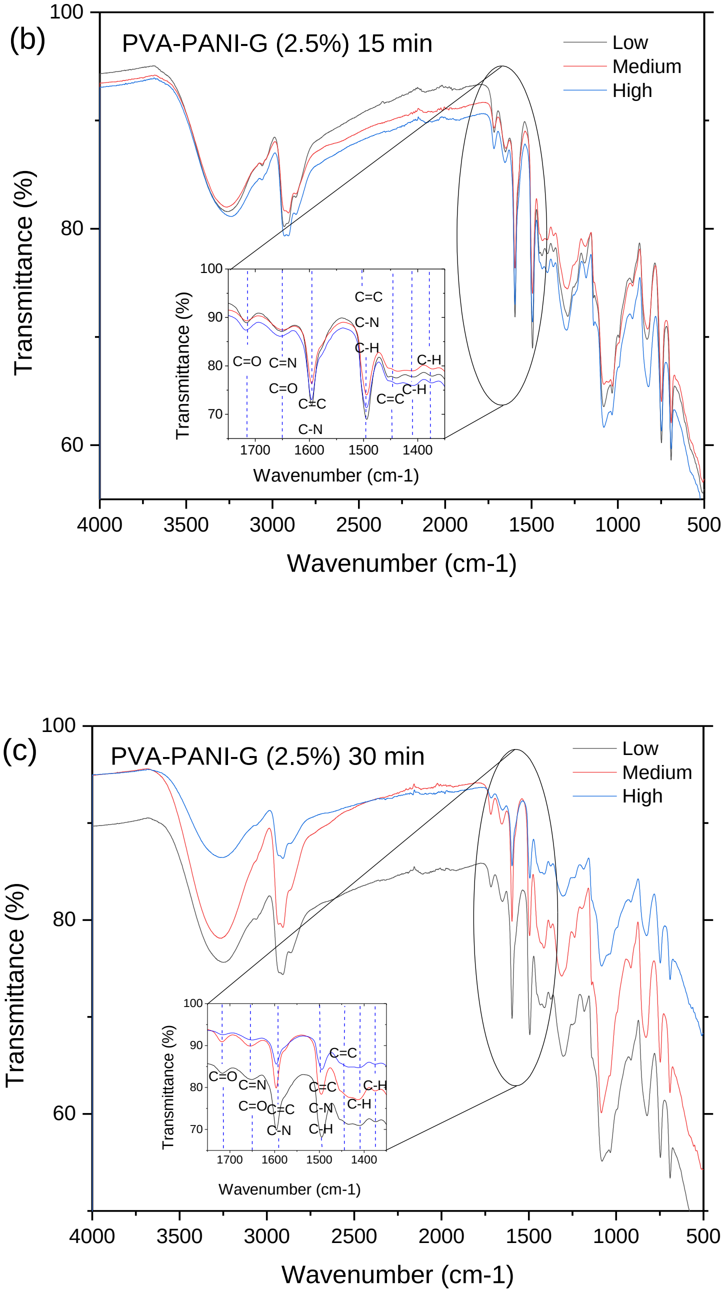

Figure S1: FTIR spectra of PVA-PANI gels at low, medium and high concentration of PANI activated with GA at 2.5% for: a) 5 min, b) 15 min, c) 30 min.

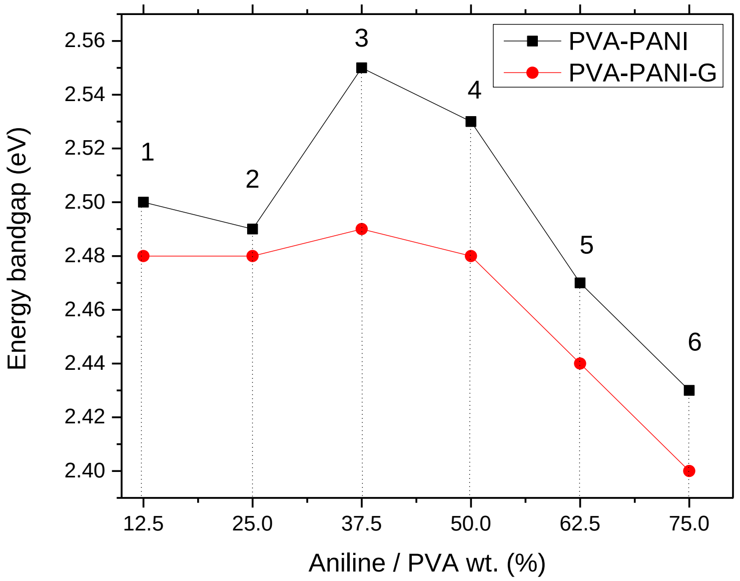

Figure S2: Bandgap variation of PV-PANI and PVA-PANI-G for different monomer concentrations.

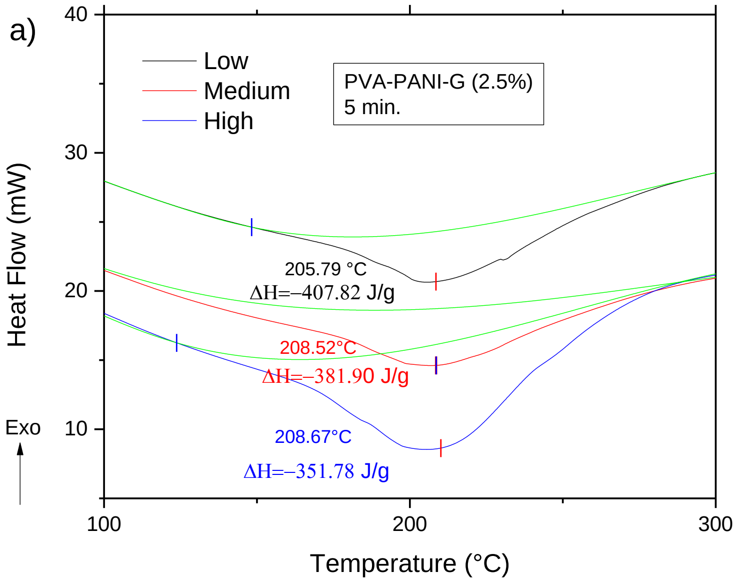

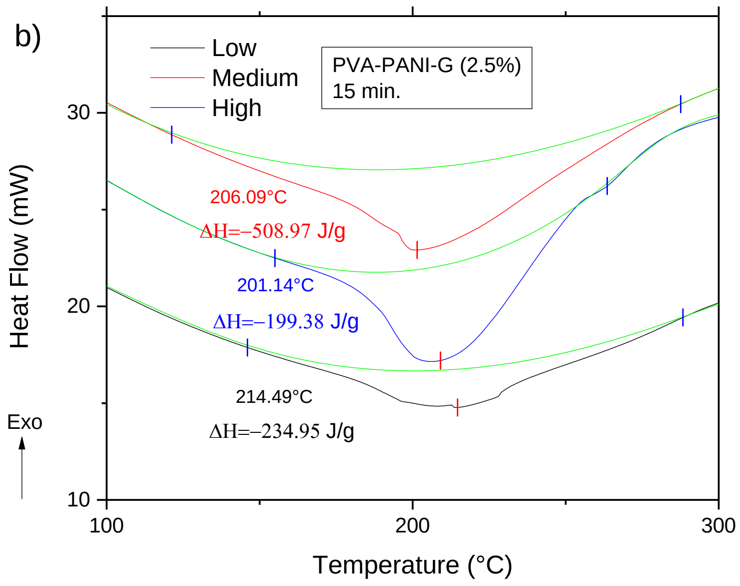

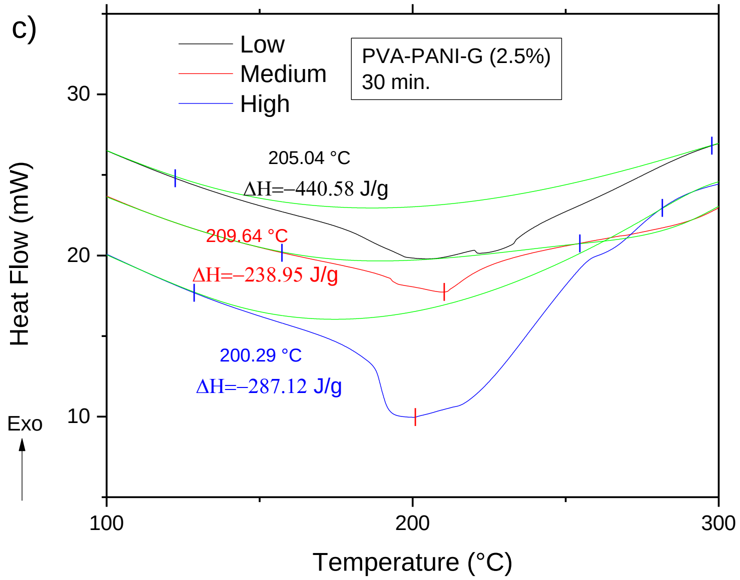

Figure S3: DSC thermogram of purified PVA-PANI gels at low, medium and high concentration of PANI activated with GA at 2.5% for: a) 5 min, b) 15 min, c) 30 min.

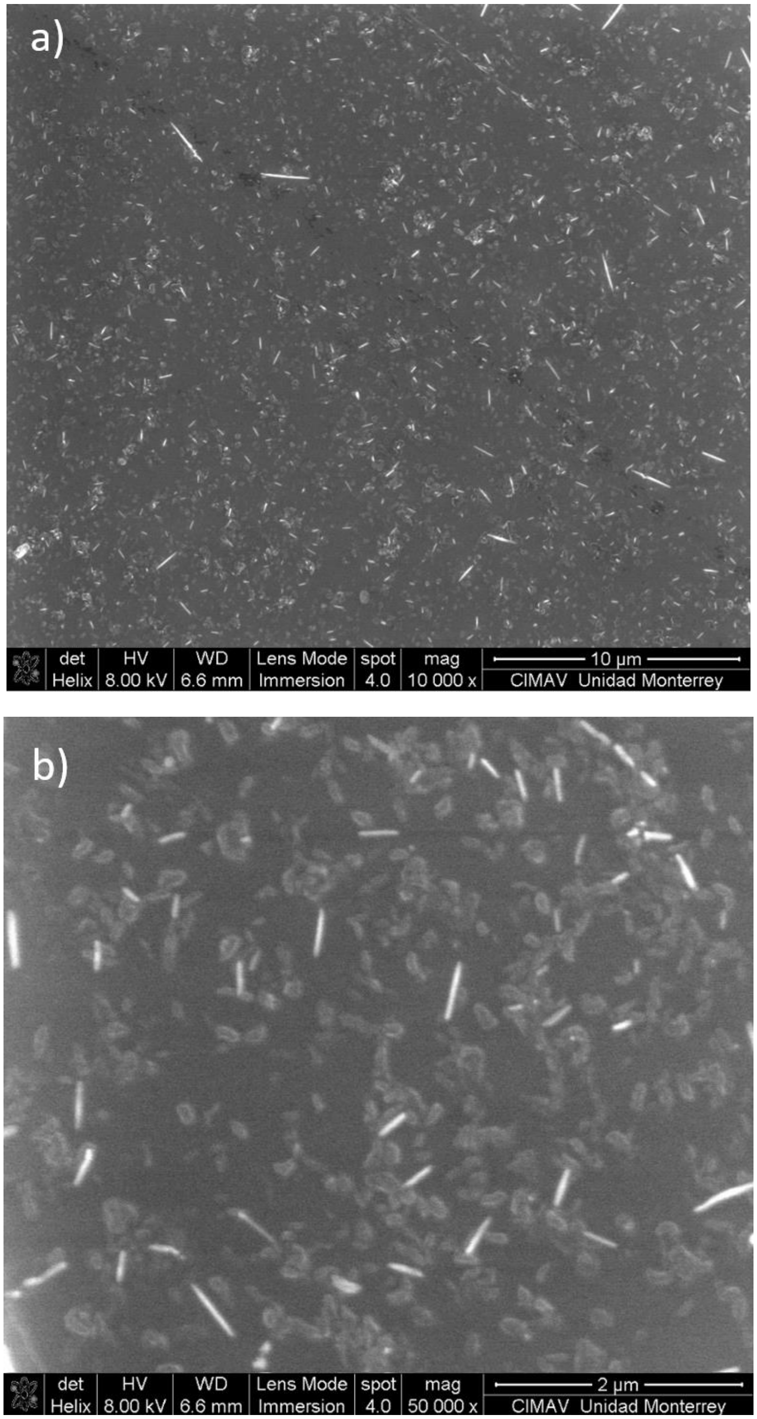
a)
det HV WD Lens Mode spot mag 10 µm
Helix 8.00 kV 6.6 mm Immersion 4.0 10 000 x CIMAV Unidad Monterrey
b)
det HV WD Lens Mode spot mag 2 µm
Helix 8.00 kV 6.6 mm Immersion 4.0 50 000 x CIMAV Unidad Monterrey

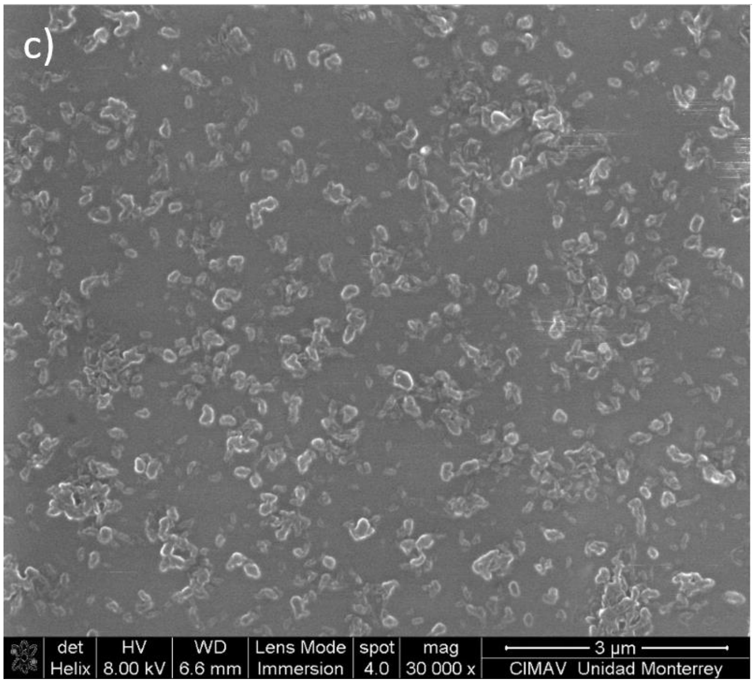

Figure S4: SEM micrographs of purified PVA-PANI thin films at a) low, b) medium and c) high concentration of aniline activated with 1% GA for 30 minutes.
